# Influence of the Selected Physical Modifier on the Dynamical Behavior of the Polymer Composites Used in the Aviation Industry

**DOI:** 10.3390/ma13235479

**Published:** 2020-12-01

**Authors:** Ewelina Kosicka, Marek Borowiec, Marcin Kowalczuk, Aneta Krzyzak, Robert Szczepaniak

**Affiliations:** 1 Department of Production Engineering, Lublin University of Technology, 20-618 Lublin, Poland; 2 Department of Applied Mechanics, Lublin University of Technology, 20-618 Lublin, Poland; m.borowiec@pollub.pl (M.B.); m.kowalczuk@pollub.pl (M.K.); 3 Department of Airframe and Engine, Military University of Aviation, 80-521 Dęblin, Poland; a.krzyzak@law.mil.pl (A.K.); r.szczepaniak@law.mil.pl (R.S.)

**Keywords:** composite material, vibrations, resonance zone

## Abstract

In this research, an analysis of polymer composite with the matrix of L285-cured hardener H286 and six reinforcement layers of carbon fabric GG 280 T was provided. It involved a comparison of the dynamical behavior responses for three cases of composite structures in the context of the presence of the mass share modifier. The samples with the addition of a physical modifier with varying mass percentages were investigated by being subjected to dynamic tests with specific parameters, i.e., constant excitation amplitude and vibration frequency in the vicinity of the base resonance zone. The analysis allowed for indicating the relationship between the composition of the prepared composites and their dynamic response via stiffness characteristics. In addition, the investigation resulted in determining the range of harmful dynamical operating conditions, which may contribute to damage to the composite structures.

## 1. Introduction

Currently, the great interest of the manufacturing industry is focused on the possibility of composite material applications [1,2], which often replace those in previous use. Activities at various levels, which bring tangible benefits in terms of the reliability of the target products, are being conducted both in the field of the manufacturing process (for example, through constantly improving machining-related aspects [3,4,5,6,7]), as well as based on the analysis of process-related operations [8,9,10,11], while simultaneously assessing the occurrence of potential threats. The aforementioned methods aim at increasing the operational reliability and efficiency of the manufactured components, and ultimately, those of the entire end product; this includes all kinds of research work focused on introducing modifications of the material composition, which impacts the observed enhancement of their properties (e.g., mechanical [12,13,14] or tribological [15,16,17,18,19,20,21,22]).

The source literature [23,24,25,26,27,28,29,30,31,32,33] describes structural composites, with their strength properties playing a key role, as well as composites with specific features (such as electrical, magnetic, thermal, and optical properties). Numerous examples of their applications indicate the need for the components made using them to satisfy stringent structural requirements. This can be evidenced by their application in demanding areas, which include aeronautics (composites used as, e.g., radar absorbing materials (RAMs) in the manufacturing of stealth aircraft) [34], wind industry (where composites are used in wind turbines) [35], or the automotive industry (the automotive market is characterized by a wide range of applications for composites in, among others, drive mechanisms and suspension, braking and exhaust systems, as well as body parts [36]).

When considering the current situation of composite materials on the global market relative to traditional materials, such as steel or aluminum, in 2009, they accounted for 5.5% of the total production, while three years later, the figure had risen to 16% [37].

According to the analysts of the German Federation of Reinforced Polymers AVK (Industrievereinigung Verstärkte Kunststoffe), the composite production growth observed in Europe has been a result of the activities by their largest consumers, who represent mainly the transport and construction industries [38]. It is estimated that the extraordinary growth in composite applications in the years 2019–2024, in comparison to the previous years, will be experienced in the transport, aviation, and wind industries [39]. This is associated with the future modification of the composite compositions and the undertaking of numerous research studies that enable determining the relationships between the applied modifiers (physical and chemical), the manufacturing methodology, and the mechanical properties.

**Figure 1:**
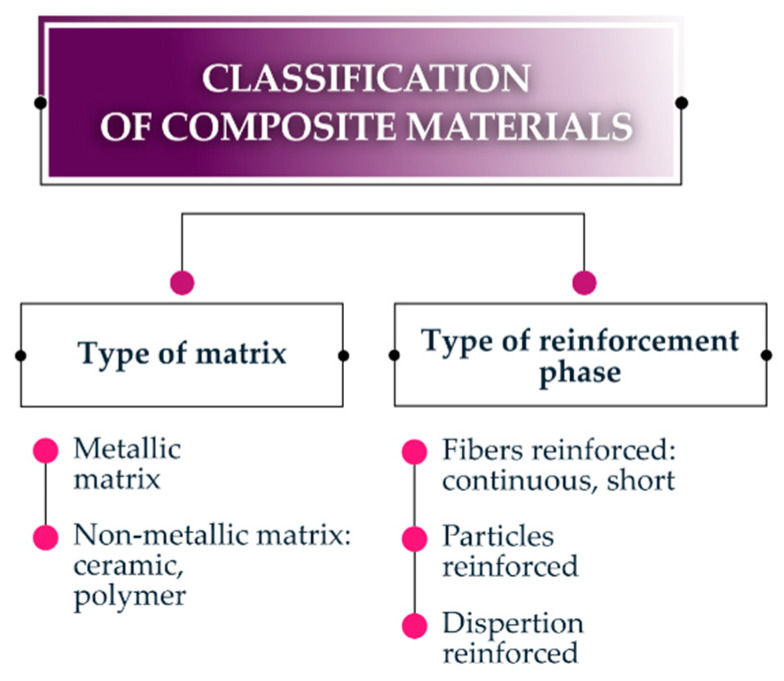
Classification of composite materials (source: own studies).

Since the obtained composite properties depend on the type of used matrix and reinforcing phase, their basic classification can be made based on the diagram below (Figure 1).

When referring to the issue of composite materials classification, one cannot ignore the structural division of the composites themselves (Figure 1. The following composites are categorized [40,41] into layered and fibrous composites, where fibrous composites use fiber (continuous or short) reinforcement, with a diameter of a fraction of a micrometer up to several hundred micrometers, where the volume fraction ranges from several percent to 70%, as well as grid, woven, and knitted and “quasi-knitted fabric,” which is reinforced with particles (also dispersive, with a minimum size on the order of 0.01–1 µm, and a volume fraction from 2 to 25%).

The application of various materials as a matrix or reinforcement results in an unlimited possibility of modifying composite properties. This has affected the growth in the interest in these materials, as observed in global industry trends. The conducted research on developing high-strength and highly modular structures, while reducing the specific weight of the composites, opens up new prospects of a bolder utilization of the composites in various sectors of the economy.

The main motivation behind developing new materials for the aviation industry is reducing aircraft weight, extending the service life of the parts, and improving fuel efficiency, thus reducing aircraft operating costs [41]. Therefore, they can be found in both the secondary and primary structures of an aircraft. Secondary structures include cabins, floorboards, or seats, but it is the primary structures that constitute the primary market for aviation composite materials [42].

The most popular examples of implementing composites in an aircraft structure include the Airbus A380 and Boeing 787 Dreamliner. Composite materials account for 25% of the former aircraft’s mass. Though it should be noted that as much as 22% of that mass is in the form of fibrous composites with an epoxy resin matrix with carbon fiber reinforced polymer (CFRP) reinforcement, which by ensuring high structural stiffness with a density that is lower than that of aluminum, enables the attainment of the lower weight of the end product [43,44].

The second aircraft, the Boeing 787, besides the use of aluminum, titanium, steel, and other metals, contains 32,000 kg of CFRP composites, which constitute 50% of the aircraft’s weight [44] (the carbon fibers alone weigh 23,000 kg). They are used to construct the fuselage, wings, tail, doors, and interior [45]. The fact that it is the composite materials that provide the wings with an aerodynamic structure, which enables reduced fuel consumption while improving the climb performance and shortening the shape length is noteworthy since such is impossible to achieve in the case of metal wings [46,47,48,49].

**Figure 2:**
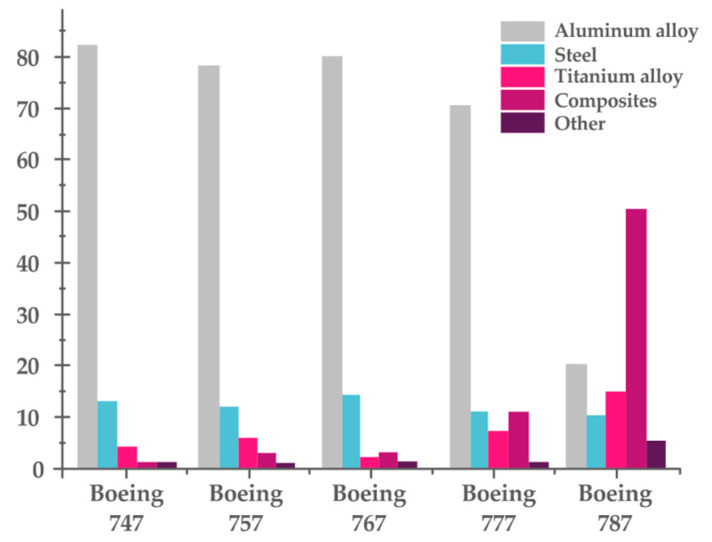
Materials used in Boeing 747, 757, 767, 777, and 787(source: own studies).

Boeing’s intensified utilization of composite materials is best illustrated by the graph below (Figure 2), which shows a shift in the trend of previously used materials for manufacturing previous aircraft models.

When mentioning the applications of aviation composite materials in advanced technologies, such as those utilized in the manufacture of the structures of the largest passenger aircraft, one must acknowledge their use in the ultralight aircraft dedicated to amateur flights. This is mainly due to the regulations governing the maximum permissible weight of these aircraft [50]. At this point, the issue of structural materials used to produce aircraft with a maximum weight of 2000 kg should be highlighted [51]. As has been observed, composite materials (including those reinforced with carbon fibers), are most often found in ultralight aircraft, where they are used mainly for the production of wings, ensuring improved performance and flight economics [52].

The source literature analysis regarding the materials used in the aviation industry has shown that carbon fibers are often used as reinforcement in polymer composites. They are mainly obtained as a result of polyacrylonitrile pyrolysis, while their properties are shaped through the careful use of manufacturing parameters [53]. Their indicated advantages often include thermal and chemical resistance, low density, and good thermal and electrical conductivity, and their application as friction materials enables obtaining a low friction coefficient [54]. Furthermore, these fibers exhibit low X-ray radiation absorption, as well as the ability to dampen vibrations, which has advantages in many fields of application. From a global perspective, this is the reason behind their wide use for the manufacturing of composite materials.

It should also be mentioned that specifying the fabric used for the produced composite material should also include the number of applied layers or the orientation of subsequent reinforcement layers. This influences the obtained composite’s properties; for example [55], the arrangement of subsequent reinforcement layers at angles of 0°, ±45°, and 90° enables impacting the directional properties of the composite and ensures properties similar to an isotropic material. Meanwhile [56], if the direction determining the fiber orientation changes from 0 to 90°, the Young’s modulus is reduced (from 260 MPa to 10 MPa). The highest Kirchhoff modulus value can, however, be obtained for a fiber orientation at an angle of 45° (the lowest for an angle equal to 0 and 90°).

It is obvious that the composite preparation method differs depending on the used materials, as well as recommendations arising from the intended use of a produced composite. Attempts to optimize the fabric layer arrangement configuration in polymer composites were referred to in the source literature, which emphasized the complexity of fabric-reinforced composite material strength analysis due to anisotropy [57]. Based on the previous research involving the observation of the mechanical properties of composites and a thorough review of the literature, it becomes possible to conduct a wide range of simulations that enable determining the impact of reinforcement orientation angles on the mechanical properties of a prepared composite. Experimental verification usually confirms the results obtained in the course of a simulation process. Furthermore, the impact of the used reinforcement layers, fiber composition, and arrangement angles on the mechanical properties of polymer composites constitute a research area for some scientists [56].

Therefore, it is worth emphasizing that the characteristic properties are obtained for a given composite, and any introduced modifications of its composition or execution entail the need to conduct research on specifying the obtained properties.

It is also obvious that the composite preparation method varies: it depends on the applied materials, as well as the recommendations from the intended use of the composite. Attempts to optimize the configuration of fabric layers in polymer composites are referred to in the literature, where the complexity of the strength analysis of composite materials reinforced with fabrics is emphasized due to anisotropy [57]. This is comprehensible due to the need for avoiding the resonance phenomenon, which is harmful to complex systems while vibrating. There are many scientific activities in the literature that are focused on the field of vibration phenomena and determining the natural frequencies of polymer composites with various applied fabrics and additives [58,59,60,61,62,63]. One of the examples presented in the literature is the PZL SW-4 helicopter, which is reported in [64]. The authors recorded a frequency spectrum taken from the vertical stabilizer while the helicopter was flying horizontally at a constant velocity of 200 km/h at 1000 m altitude. The measurements reported a range of frequencies and amplitudes that enabled identifying the important design variables. Based on the results obtained, while the helicopter was flying, the noticeable vibration levels occurred at frequencies of 22, 30, and 45 Hz. There are sensitive frequencies of such an aircraft structure and the dimensions of the material samples are worth recording for the purpose of analyzing its dynamics around this frequency range.

This indicates that there are changes resulting from the composition of the composite. Dinesh et al. [58] reported wider considerations in this area. The parallel tribological studies of designed composites, which will have an application dimension in the future, demanded that the authors determine the dynamical behavior responses, especially the vibration characteristics. This is addressed in this article

## 2. Measurements Methodology

### 2.1. Composite Beams

This research concerned polymer composites. The manufacturing of composites was carried outusing the hand lamination method. The specimens were made of certified carbon fabric GG 280 T (G.Angeloni srl, Quarto d’Altino, Italy) (twill 2/2, fiber 3K 200 tex, 220 g/m^2^) and an epoxy resin with the trade name L285, which includes the hardener H285 (German Luftfahrt-Bundesamtal, Havel Composites, Přáslavice, Czech Republic). The usage of such materials was done to mirror aircraft composites, specifically the ones used in the PZL SW-4 helicopter. The physical modifier was white corundum (Al_2_O_3_) with a purity of 99% and granularity designated as F280 (abrasive grain division according to the FEPA 42-2:2006 [65] standards). In order to observe the impact of the added modifier, three sets of samples were made, one with a 5% modifier content, one with a 15% modifier content, and a set without white corundum (0%). The corundum was added before the resin saturation. The composite consisted of six fabric layers, where the layout was arranged in the configuration: 0°/22.5°/45°/0°/22.5°/45°. The fiber configurations were determined using laminates applied in the aviation industry. The hand laminating method was carried out in a specific way. First, the hand layup was prepared; the consecutive fabric layers were arranged in the desired configuration, where each was impregnated by resin. In order to make sure that the composites had identical thicknesses and properties in the whole area prior to curing, the structure was pressed by means of the hydraulic press PDM–50S Mecamaq (Mecamaq, Lleida, Spain) at the pressure level of 2.5 MPa (duration: 24 h). The occurrence of filler agglomeration was eliminated by using ultrasonic waves.

The samples for dynamical investigation were prepared by cutting a 2.4 mm thick plate in the shape of a beam with the dimensions of 200 × 10 mm by means of an abrasive water jet system traveling in a perpendicular direction [66]. The final dimensions of the samples were measured as 200 × 10 × 2.4 mm (see Table 1), where the frequency of the vibrations was investigated in the two consecutive resonance zones.

### 2.2. The Vibrations of the Beam in the First Resonance Zone

**Figure 3:**
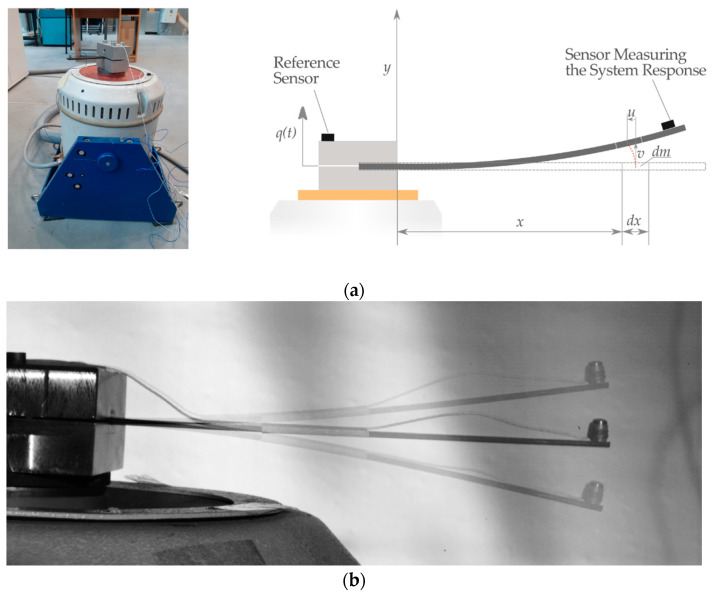
(**a**) The experimental setup and the scheme of the vertically excited composite beam and (**b**) the captured views of the beam vibrations in the first resonance zone.

In this section, the differential equation of the beam motion is developed and the experimental approach is discussed. The governing equation is presented using the Euler–Bernoulli beam theory. It was assumed that the thickness of the beam was small compared with the length; thus, the shear deformation effects could be neglected. Figure 3a shows the beam as a horizontally excited cantilever of length *L*, and in Figure 3b, its response positions.

The assumptions for the mathematical model are as follows: an arbitrary mass-point of the beam *dm* undergoes horizontal and vertical displacements *u(t*) and *q*(*t*) *+ v*(*t*), and rotates *φ*(*t*) around the *z*-axis due to system excitation from the shaker payload. The inertia from the sensor was included in the model. The differential equation of the beam motion was derived by means of the Lagrangian approach [67], as shown in Equation (1):

(1) $$\frac{d}{dt}\left(\frac{\partial T}{\partial \dot{v}}\right)-\frac{\partial T}{\partial v}+\frac{\partial \Pi }{\partial v}=0,$$

where the kinetic and potential energies are denoted by *T* and *Π*, respectively, and for an infinitely small mass *dm* of the beam, they are as follows (Equation (2)):

(2) $$T=\frac{1}{2}\rho A\int_{0}^{L}\left[\dot{u}{\left(x,t\right)}^{2}+{\left(\dot{v}\left(x,t\right)+\dot{q}\left(t\right)\right)}^{2}\right]dx+\frac{1}{2}\int_{0}^{L}J\omega^{2}dx+\frac{1}{2}M\left[{\dot{u}}_{M}{\left(t\right)}^{2}+{\left({\dot{v}}_{M}\left(t\right)+\dot{q}\left(t\right)\right)}^{2}\right],\;\Pi =\frac{1}{2}EI\overset{L}{\underset{0}{\int }}\kappa {\left(x,t\right)}^{2}dx.$$

The symbols employed in the energies formulae are as follows: *ρ* stands for the density of the beam mass; *A* is the cross-section of the beam; *J* denotes the principal mass moment of inertia per unit length of the beam; *ω* is the angular velocity around the *z*-axis, which is perpendicular to the vibration plane *xy*; *M* is the mass of the attached the accelerometer and is denoted “tip mass”; *EI* denotes the flexural rigidity of the bent part of the beam; *κ* is the curvature of the beam.

The displacements of the beam are expressed by the function of the beam deflection in the form $v\left(x,t\right)=\psi \left(x\right)v\left(t\right)$, where $\psi \left(x\right)$ describes the geometrical beam deformation (Equation (3)):

(3) $$\psi \left(x\right)=\cosh\left(\lambda \frac{x}{L}\right)-\cos\left(\lambda \frac{x}{L}\right)-\eta \left(\sinh\left(\lambda \frac{x}{L}\right)-\sin\left(\lambda \frac{x}{L}\right)\right),\;\eta =\frac{\sinh\lambda -\sin\lambda +\lambda \frac{M}{\rho AL}\left(\cosh\lambda -\cos\lambda \right)}{\cosh\lambda +\cos\lambda +\lambda \frac{M}{\rho AL}\left(\sinh\lambda -\sin\lambda \right)},$$

where *λ* corresponds to the consecutive modes of the beam and can be calculated from the transcendental equation:

(4) $$1+\cosh\lambda \cos\lambda +\lambda \frac{M}{\rho AL}\left(\cos\lambda \sinh\lambda -\sin\lambda \cosh\lambda \right)=0.$$

Calculating the consecutive derivatives of the energies parts according to Equation (1), the constants depending on the shape function for the first mode take the forms given below [68], (Equation (5)):

(5) $$N_{1}=\int_{0}^{l}\psi {\left(x\right)}^{2}dx=0.25L\;\left(m\right),\;\;\;\;N_{2}=\int_{0}^{l}\psi \left(x\right)ds=0.39L\;\left(m\right),\;\;\;N_{3}=\int_{0}^{l}(\int_{0}^{s}{\left(\psi ’{\left(x\right)}^{2}dx\right)}^{2}dx=0.28/L\;\left(m^{-1}\right),\;N_{4}=\int_{0}^{l}\psi ’{\left(x\right)}^{2}ds=1.17/L\;\;\left(m^{-1}\right),\;N_{6}=\int_{0}^{l}\psi ’’{\left(x\right)}^{2}dx=3.05/L^{3}\;\left(m^{-3}\right),\;\;\;\;\;\;N_{9}=\int_{0}^{l}\psi ’{\left(x\right)}^{4}ds=1.86/L^{3}\;\left(m^{-3}\right).$$

Introducing the corresponding derivatives of the energies to Equation (1) and neglecting the third- and higher-order terms of $v\left(t\right)$ and $\dot{v}\left(t\right)$, the differential equation of motion reached the form of Equation (6):

(6) $$\begin{array}{ll} {}[\varrho AN_{1}+\frac{h^{2}}{12}\rho AN_{4} & +M+\left(\rho AN_{3}+\frac{h^{2}}{12}\rho AN_{9}+MN_{4}^{2}\right)v{\left(t\right)}^{2}]\ddot{v}\left(t\right)\\ & +\left(\rho AN_{3}+\frac{h^{2}}{12}\rho AN_{9}+MN_{4}^{2}\right)\dot{v}{\left(t\right)}^{2}v\left(t\right)+EIN_{6}v\left(t\right)=-\left(\varrho AN_{2}+M\right)\ddot{q}\left(t\right). \end{array}$$

The linearized equation of motion for the free response about the equilibrium position (Equation (7)) allowed for estimating the natural frequency of the beam:

(7) $$\left[\varrho AN_{1}+\frac{h^{2}}{12}\rho AN_{4}+M\right]\ddot{v}\left(t\right)+EIN_{6}v\left(t\right)=0,\;f_{n}=\frac{1}{2\pi }\sqrt{\frac{EIN_{6}}{\varrho A\left(N_{1}+\frac{h^{2}}{12}N_{4}+M\right)}},$$

**Figure 4:**
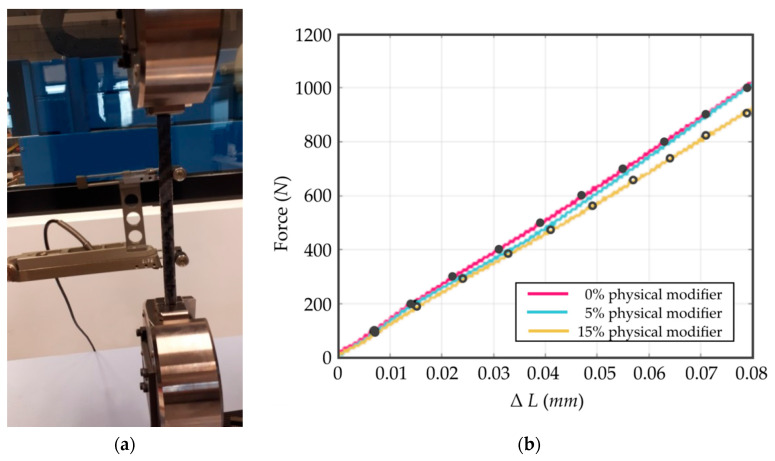
(**a**) Evaluating the mechanical properties of the composite material using a Shimadzu universal testing machine and (**b**) the characteristics of the material for the modulus of elasticity, which resulted from the tensile testing for three cases of the share modifier included in the composite.

The parameters of the tested material are listed in Table 1. The Young’s modulus was estimated experimentally using tensile tests on a Shimadzu AGS-X 10 kN stand, as shown in Figure 4a. The results are plotted in Figure 4b.

Choosing ten points from the tensile testing, the Young’s modulus was estimated using Equation (8) and was equal *E_0%_* = *E_5%_* = 42,398 MPa for the composite without the physical modifier of aluminum oxide (0%), and for the composite including the aluminum oxide at 5%. The case of the physical modifier at 15% differed, where the Young’s modulus reached *E_15%_* = 38,297 MPa.

(8) $$E_{i}=\frac{\left(P_{i+1}-P_{i}\right)L}{\left(\Delta L_{i+1}-\Delta L_{i}\right)A}$$

For isotropic properties of the samples’ structure, using the parameters from Table 1, the natural frequencies for the first mode from Equation (7) reached values that corresponded to the material without the physical modifier, *f_n0%_ =* 41 Hz, but for the material with a 5% physical modifier, *f_n5%_* = 39 Hz, and in the third case, for the material with a 15% physical modifier, *f_n15%_* = 37 Hz.

**Figure 5:**
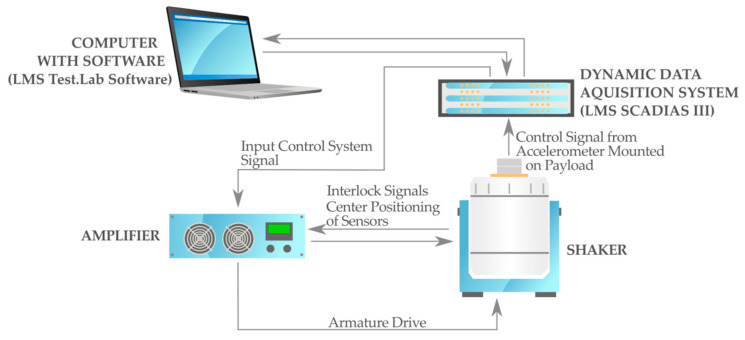
The laboratory armature of the electro-dynamical vibration exciter.

The dynamical behavior of the composite beam was tested in two different experiments. The electromagnetic shaker system TIRAvib 50101 included in the armature shown in Figure 5 was the first conducted test.

The measurement system was supported by the dynamic LMS Scadias III controller and the Test.Lab 14A software (LMS INTERNATIONAL, Leuven, Belgium) [69]. The armature reproduced specified environmental conditions for the beam structure. For comparable responses of the beam vibrations, the excitations were realized using a sinusoidal input signal at a constant level that corresponds to a gravitational acceleration of 1g. The behavior of the composite beam was recorded by two acceleration sensors that were designed for the assumed frequency range (Figure 3).

**Figure 6:**
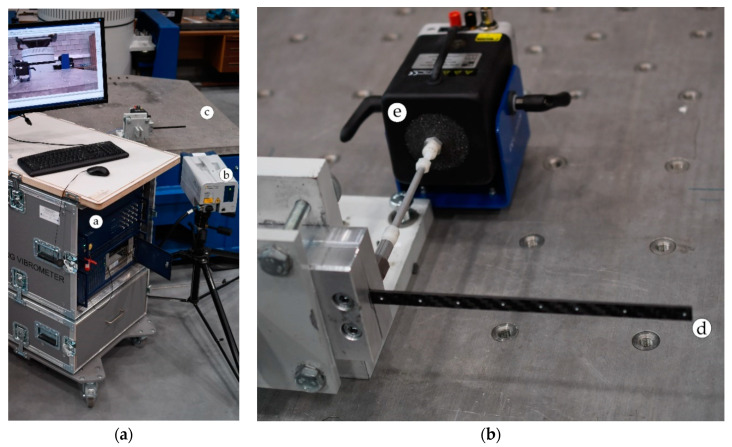
Experimental setup for the laser vibrometer tests: (**a**) view of the setup of the laser vibrometer PSV-500 camera system and (**b**) dedicated grip with the specimen (close-up view). Parts of the setup: a—mobile workstation, b—PSV scanning head, c—anti-vibrational table, d—specimen, and e—electrodynamic shaker.

The second experiment was conducted using the laser vibrometer shown in Figure 6.

The setup for the experiment consisted of the laser vibrometer PSV-500 camera system (Figure 6a, marks a and b), a specimen shown in Figure 6b, mark d, which was mounted using a dedicated grip to an anti-vibrational TIRA TGT MO 48XL slip table (Figure 6a, mark c), which isolated the undesirable vibrations coming from the ground. Excitation was delivered using a SmartShaker Mini model K2007E01 electrodynamic shaker (Figure 6b, mark e).

During the experiment using the scanning vibrometer, the instantaneous 3D coordinates of the previously set reflective markers were traced by the laser scanning head (Figure 6a, mark b) to capture the dynamical response of the specimen. Data collected in such a way was based on the Doppler effect, correlating the measured frequencies of the laser beam emitted and reflected back to the camera, as well as the specimen velocities relative to the laser ray. The received signal was converted into a frequency response function using the fast Fourier transform (FFT), from which, the natural frequencies and mode shapes of the tested structure were calculated.

**Figure 7:**
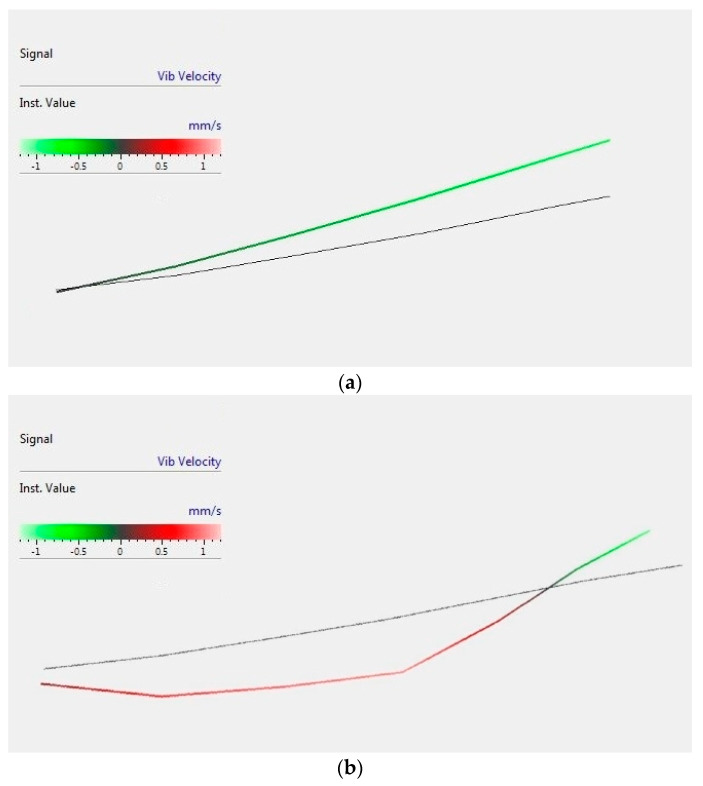
(**a**) First and (**b**) second bending mode shapes obtained with the laser vibrometer.

The performed modal analysis allowed for standard frequency comparisons derived from the FFT analysis, as well as mode shape studies. In the following figure, the first and second bending modes extracted from the laser vibrometer test are presented (Figure 7).

## 3. Results

**Figure 8:**
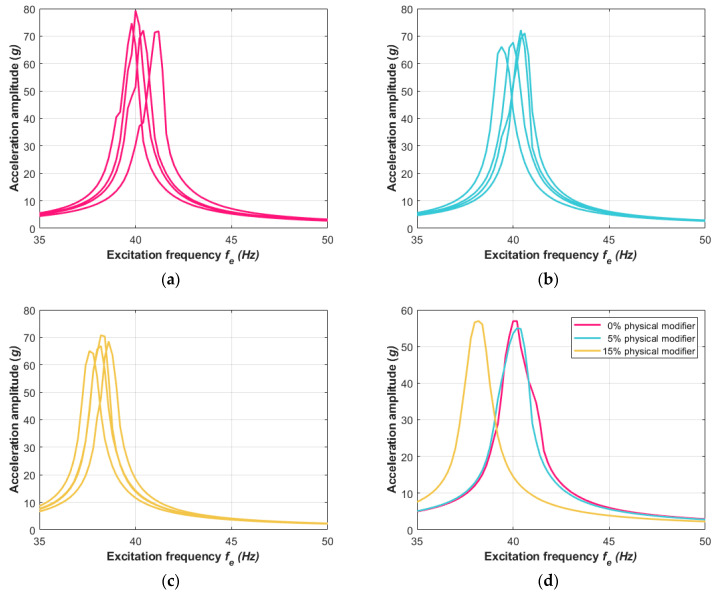
The response acceleration amplitude of the composite beams via excitation with (**a**) 0%, (**b**) 5%, and (**c**) 15% physical modifiers. (**d**) The mean values of the selected characteristics for each case of physical modifier are also included.

The experimental measurements were carried out for the three sets of the composite samples. Each set differed in terms of the mass share of the physical modifier; two cases of the beam samples included 5% and 15% mass share modifiers. The proposed percentage mass shares of the modifier were determined on the basis of optimization carried out concerning previously obtained results of mechanical tests. These dynamical investigation results were compared to those of the composite beam where the mass share of the physical modifier had not been included. First, the experiment using the TIRA shaker was carried out. The vibration conditions were fixed for harmonic excitation at a constant acceleration corresponding to 1*g* of gravitational acceleration. The investigations via the vibration tests were provided using four beam samples for each case. These results are presented in Figure 8a–c. In Figure 8d, the averaged results of three cases regarding the shared modifier in the material are shown.

The previously selected set of four beams for each physical modifier consistency was tested using the second method of experimentation. The boundary conditions were identical thanks to the employment of the very same dedicated grip; however, this method of measurement was contactless; therefore, there was no need to apply the accelerometer at the free end of the beam. The applied excitation was harmonic, but the excitation apparatus was a different type of shaker. This did not change the qualitative conditions of the test; only quantitative changes occurred.

**Figure 9:**
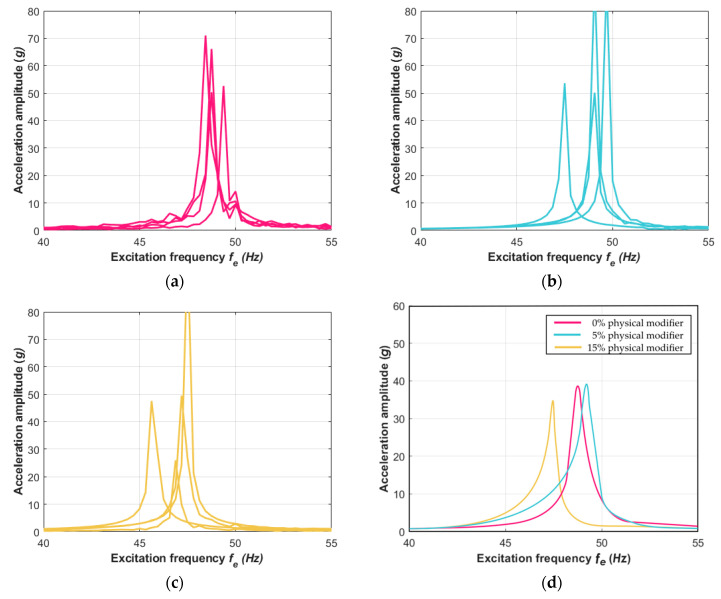
Second experiment method outcomes of the tests of samples (**a**) without the physical modifier and the samples that included (**b**) 5% and (**c**) 15% of the physical modifier, respectively. (**d**) The mean values of selected characteristics for each case of physical modifier are also included.

**Figure 10:**
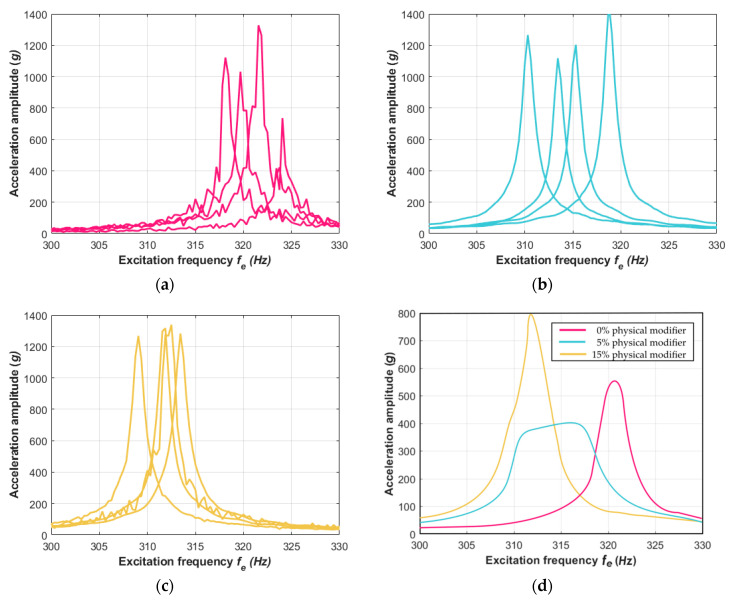
Second bending mode obtained from the laser vibrometer experiment of samples (**a**) without the mass share modifier and that included (**b**) 5% and (**c**) 15% of the physical modifier. (**d**) The mean values of selected characteristics for each case of each physical modifier are also included.

The amplitude–frequency responses reached their resonance peaks in zones according to the frequencies *f_n_* that were analytically found using Equation (7). The averaged acceleration amplitudes that are shown in Figure 8d and Figure 9d clearly point out the influence of the physical modifier on the dynamics of the composite structure. The case of the material without the modifier reached the resonance frequency *f_n_* at around 40 Hz for the TIRA shaker test and 49 Hz with the laser vibrometer test. The next set of beam samples, which included a slight 5% physical modifier, returned a similar dynamic response and *f_n_* was also around 40 Hz and 49 Hz for their respective experiments. For the third composite material set with a 15% physical modifier, the resonance zone moved to lower values and reached the frequency *f_n_* at around 38 Hz and 46 Hz, respectively. This revealed that increasing the share of the physical modifier resulted in decreasing the stiffness of the vibrating beams. This phenomenon is similar to that found in vibrations of structures created of the same composite plate but in directions that are perpendicular to each other, as discussed in [65]. The dynamical responses of the tested samples in the second bending mode demonstrated the influence of the share modifier on the composite features more clearly. This is presented in Figure 10a–d, where the amplitude–frequency characteristics are reported in the vicinity of the second mode shape. The vibrations at higher frequencies around 310 Hz definitely confirmed that the share modifier decreased the stiffness of the composite structure. The response at lower vibration frequencies for 0% and 5% physical modifiers revealed hardly any difference in their resonance peaks (Figure 8d and Figure 9d), but the difference is clearly exposed in the corresponding Figure 10d.

The maximal output amplitudes accordingly moved down as the modifier share was gradually increased. These features of the analyzed composite structures give a wide range of opportunities in the manufacturing processes of specific aircraft elements. The stiffness of the similar geometric shape of parts can be easily modified for a given technological purpose.

## 4. Conclusions

The obtained results indicate that there was an impact of the amount of added modifier on the dynamical behavior of the samples. This was revealed by the amplitude–frequency response values while the composite structure was vibrating in its resonance range. The modifiers allowed for changing the stiffness of the structure without modifying the geometrical shapes or sizes and a drop in resonance frequency was observed when comparing the results of beams without a modifier to those containing a modifier that made up 15% of the total mass of the specimen. Thus, was been reported that increasing the mass share of the physical modifier resulted in the increase of the material’s compliance. The scientific values found in this study indicate the existence of a relationship between the change of the share of a physical modifier in a polymer composite reinforced with carbon fabric.

The demonstrated methods of the experimental and analytical approaches had consistent results for the exhibited beams; therefore, the methods are recommended for testing similar specimens. The decrease in stiffness caused by increasing the modifier share in the total mass of the specimens was more apparent in the second bending mode. The experiments revealed that the modifier had a greater impact on higher modes; therefore, in applications where the second bending mode is the most hazardous for the system, less modifier (5%) can be used to obtain the expected shift in resonance frequency. In the case where the first mode is of paramount concern, it is suggested to add 15% of the mass as the modifier to avoid the dangerous frequency region. In the authors’ final investigations, there was a verified influence of the percentage mass share of the physical modifier on the tribological wear. The optimization of the composite material compositions as the criteria to be improved regarding anti-wear and mechanical properties is necessary for defining changes taking place in the scope of vibration analysis of these composite structures, especially in the resonance area. The results discussed in this paper are from one of this project’s stages, which was to determine the influence of physical modifiers, such as Al_2_O_3_ and SiC, on dynamic behaviors. It seems appropriate to focus additionally on the impact of the grain size of the modifiers and determine its influence on the dynamical properties of composite materials.

## Figures and Tables

**Table 1:** The parameters of the composite material beam.

Symbol and Value	Description
*ρ_0%_* = 1344 kg/m^3^	Mass density of the beam without the physical modifier
*ρ_5%_* = 1507 kg/m^3^	Mass density of the beam with 5% of the physical modifier
*ρ_15%_* = 1533 kg/m^3^	Mass density of the beam with _15%_ of the physical modifier
*E_0%_* = 42.40 GPa	Young’s modulus of the material without the physical modifier
*E_5%_* = 42.40 GPa	Young’s modulus of the material with 5%of the physical modifier
*E_15%_* = 38.30 GPa	Young’s modulus of the material with 15% of the physical modifier
*L* = 200 mm	Length of the beam
*b* = 10 mm	Width of the beam
*h* = 2.4 mm	Thickness of the beam
*A* = 24 mm^2^	Cross-section of the beam
*I* = 11.52 mm^4^	Area moment of inertia
*N_1_* =0.05 m	Constant no. 1 depends on *ψ*(*x*)
*N_4_* = 5.80 m^−1^	Constant no. 4 depends on *ψ*(*x*)
*N_6_* = 386.25 m^−3^	Constant no. 6 depends on *ψ*(*x*)
*f_n0%_* = 41 Hz	Natural frequency of the beam without the physical modifier
*f_n5%_* = 39 Hz	Natural frequency of the beam with 5% of the physical modifier
*f_n15%_* = 37 Hz	Natural frequency of the beam with 15% of the physical modifier
